# Impact of Virtual Heartfulness Meditation Program on Stress, Quality of Sleep, and Psychological Wellbeing during the COVID-19 Pandemic: A Mixed-Method Study

**DOI:** 10.3390/ijerph182111114

**Published:** 2021-10-22

**Authors:** Kunal Desai, Pratibha Gupta, Priti Parikh, Alpa Desai

**Affiliations:** 1 Department of Internal Medicine, Boonshoft School of Medicine, Wright State University, Dayton, OH 45435, USA; alpa.desai@wright.edu; 2 Food Nutrition and Health Agricultural Research Development Program, Central State University, Wilberforce, OH 45384, USA; pgupta@centralstate.edu; 3 Department of Surgery, Wright State University, Dayton, OH 45435, USA; priti.parikh@wright.edu

**Keywords:** Heartfulness meditation, stress, sleep, pandemic, psychological wellbeing, COVID-19

## Abstract

Stress and lack of quality sleep affect a large portion of the population around the globe, and the COVID-19 pandemic has genuinely brought attention to these problems. This study aimed to investigate whether using a virtual heart-based meditation program is associated with improved stress levels and quality of sleep among participants from the general population during the COVID-19 pandemic. We recruited 63 participants to receive an 8-week virtually conducted Heartfulness meditation program in a prospective pre–post single-arm intervention study from September 28 to November 22 2020. Perceived Stress Scale (PSS) and Pittsburgh Sleep Quality Index (PSQI) scores were collected at baseline, at 4 weeks, and 8 weeks. Of the 63 participants enrolled in the study, 36 (57%) completed an 8-week Heartfulness meditation program. There was a significant decrease in PSS (mean difference of 6.68 with 95% C.I. 4.89–8.47, *p* < 0.0001) and in PSQI (mean difference of 2.05 with 95% C.I. 1.03–3.07, *p* < 0.0001) between week zero and week eight, regardless of Health Care Professional status. The qualitative thematic analysis strongly supported the survey results. A significant reduction in perceived stress score and improvement in sleep quality index was noted at the end of a virtual Heartfulness meditation program. Moreover, Heartfulness meditation practice may help cultivate the quality of empathy, acceptance, and individual peace. We conclude that the effects of virtually accessible Heartfulness meditation practice need to be explored further in larger studies.

## 1. Introduction

Stress is a condition or feeling experienced when a person perceives that demands exceed the personal and social resources the individual can mobilize. It can be simplified as a demand-supply mismatch of one’s inner resources and inner or external needs. Stress is triggered by intrinsic or extrinsic factors associated with biological responses. Stress is considered a causative or contributing factor for many health problems, including hypertension, cardiovascular disease, obesity, diabetes, mental health disorders, immune dysfunction, and sleeping disorders, as cited by the previous authors [1,2]. Sleep is one of the single most important factors determining the level of stress and overall health; similar to stress, insufficient sleep or sleep disorders leads to physical and emotional health problems [3]. Therefore, stress and lack of quality sleep are considered serious public health challenges which our community face despite modern lifestyles, comforts, and technological advances. The COVID-19 pandemic has genuinely brought attention to this preexisting stress problem by making it significantly worse. A recent 2021 survey by the American Psychological Association revealed that 48% of Americans reported an increased level of stress. Approximately 67% reported sleeping more or less than they wanted to since the pandemic started [4]. The Centers for Disease Control and Prevention (CDC) has recommended several ways to cope with stress, including eating regularly, good sleep hygiene, exercise, relaxation, and meditation [5].

There are various meditation techniques, but this study focuses on Heartfulness meditation, a simple heart-based meditation practice aimed at attaining a balanced state of mind. The practice comprises a morning session focusing on relaxation and meditation, an evening rejuvenation session that involves removing emotional impressions of the day, and a session at night for a deeper connection with oneself involving a short meditation session before sleep. In addition, trainer-guided sessions are also offered. Meditation programs in general, including Heartfulness meditation, have shown a positive impact on psychological stress and well-being in the participants with specific medical or mental health needs [6,7,8,9]. However, psychological stress is experienced by a large portion of the population, especially during the COVID-19 pandemic [4]. Assessing the impact of meditation programs among the general population can be helpful to understand how best to utilize such interventions. There is vast literature assessing the impact of different meditation practices on emotional regulation, health-promoting, and prosocial behaviors. Mindfulness meditation practices have been shown to improve emotional regulation and reduce drug abuse [10]. Cross-sectional data showed that US adults who practiced mindfulness meditation are more likely to be physically active [11]. A systemic review and metanalysis of 26 randomized trials support the efficacy of meditation-based interventions for increasing empathy, compassion, and pro-social behaviors [12]. Limited qualitative data exists regarding Heartfulness meditation practice on the participants’ experiences and perceived changes in their overall attitude towards life and wellbeing. Moreover, the current literature lacks data regarding effectiveness of virtual delivery of meditation programs [7,13,14]. This study, therefore, seeks to investigate whether using a virtual heart-based meditation program is associated with improvements in stress levels and quality of sleep among participants from the general population. Moreover, we report participants’ experiences and perceived changes that are crucial in providing better insights and further improving the utilization of such practices.

## 2. Materials and Methods

### 2.1. Study Design and Data Collection

This prospective pre–post single-arm intervention study assessed the effect of an 8-week heart-based meditation on the level of stress using the Perceived Stress Scale (PSS) and quality of sleep using the Pittsburgh Sleep Quality Index (PSQI). The participants were surveyed on coronavirus disease 2019 (COVID-19)-related stress, i.e., whether or not they are worried about themselves or anyone in the family catching this infection. All the participants were asked to complete this survey and PSS and PSQI scale survey at baseline, week four, and at the end of the study period of 8 weeks.

Qualitative data on the impact of such meditation practices on overall wellbeing, interaction with others, and stress management were also collected. All participants were offered optional one-to-one virtual interview sessions, which took place within three weeks after the end of the study period that were recorded and transcribed for thematic analysis.

### 2.2. Recruitment of Participants

The study was open to the public in the United States of America from 28 September–22 November 2020. Participants were adults above 18 years of age willing to participate in the study, have a basic knowledge of the Internet, and able to follow instructions regarding email communications and accessing video conferences. Any person under medical care for depression or other mental health conditions was encouraged not to participate or only participate after discussion with his/her healthcare provider so that the study participation did not interfere with current treatment. The study participants were recruited from different parts of the United States. The information regarding the study was provided to the public through social media platforms such as Facebook using a flyer. Several local universities, healthcare institutions, and corporations were also provided information through personal and professional contacts. The website page was created by Heartfulness research institute with frequently asked questions regarding the research study and a link to a consent form. The research project was explained to willing participants, and any questions were answered before signing any informed consent.

### 2.3. Intervention

#### 2.3.1. Orientation and Education Sessions

The primary investigator conducted a live virtual orientation session on the aspects of the study and the structure of the meditation protocol before the start of the study. Participants were also briefed about expectations during meditation sessions and were offered the contact details of the trainer for any further questions. They were provided with educational material via email consisting of essential information regarding Heartfulness meditation practice. The primary investigator conducted six live virtual education sessions via PowerPoint presentations every weekend during the first 6 weeks of the study period on these topics in the following order: benefits of meditation, challenges and tips to improve meditation practice, learn rejuvenation technique, learn bedtime prayer meditation, heartfulness practice overview, and formal questions and answers. These sessions were recorded, and links to video recordings were shared with all study participants to review at their convenience if unable to attend live sessions.

#### 2.3.2. Meditation with a Heartfulness Trainer

I. The participants were recommended to attend a minimum of two out of a total of eight virtual trainer-guided group Heartfulness relaxation and meditation sessions each week. These sessions, conducted by one of the authors and a Heartfulness trainer (KD), included 5–7 min of relaxation followed by 20 min of meditation. Heartfulness meditation practice asks participants to gently focus their attention, with eyes closed on the source of light within the heart. Rather than trying to visualize this, participants were asked to simply tune in to their hearts and be open to any experience that they may have. If their mind wanders, participants were advised to redirect toward the heart gently [9].
II. The participants were also provided instructions on using the phone application called ‘HeartsApp’ on their phones. They could connect as an anonymous seeker with a Heartfulness trainer through the application and meditate without any audiovisual interaction.

#### 2.3.3. Self-Practice 

In addition to attending a minimum of two out of a total of eight virtual trainer-guided group Heartfulness relaxation and meditation sessions each week, they were also suggested self-practices to the best of their abilities.

I. Heartfulness relaxation followed by meditation preferably in the morning or any other convenient time for about 20 min with the same technique as guided group sessions. They may listen to Heartfulness relaxation using online resources on www.heartfulness.org.
II. The following two self-practices were advised to the participants after completing education sessions for each technique.
  - Heartfulness Rejuvenation: An evening practice of rejuvenation lasting 15 min was recommended where they imagine that stress and heaviness were leaving the body through the back in the form of smoke or vapor. These heaviness and stress were to be replaced by a flow of purity, lightness, and freshness.
  - Bedtime Prayer meditation: Participants were recommended to prayerfully contemplate the day’s events for self-introspection and meditate on the source of light within the heart for about 5 min before sleeping.

Participants were also provided with a practice tracker sheet to record attendance in the group meditation sessions, self-practice, and meditation using Hearts App. The practice tracker sheets were asked to be submitted every 2–3 weeks. The investigators recorded group meditation session attendance.

#### 2.3.4. Statistical Analysis

Basic demographic information, including age, gender, and level of physical activity, were collected. Participants were evaluated for their perceived stress and sleep quality using the measurement tools with well-established reliability and validity, such as Perceived Stress Scale (PSS) and the Pittsburgh Sleep Quality Index (PSQI). In order to determine if there were any significant decreases in these variables over the course of the study, each of them was analyzed via a repeated measures ANOVA. The repeated measure was the time (0 weeks, 4 weeks, and 8 weeks). An independent variable was included for whether the respondent was a Health Care Professional (Yes or No) out of concern that they may be experiencing a higher level of stress while working during the COVID-19 pandemic. The presence of an interaction between Time and Health Care Professional was investigated first. A significant interaction would indicate any changes in the response variables over time are not always constant for Health Care Professionals and non-Health Care Professionals. If the interaction term was not significant, it was removed, and the main effects of Time and Health Care Professional were assessed directly. Post hoc multiple comparisons were made using Tukey’s multiple comparison procedure. A level of significance of α = 0.05 was used throughout to assess statistical significance, and SAS version 9.4 (SAS Institute, Inc., Cary, NC, USA) was used for all analyses. The qualitative data on interviews, on the other hand, were analyzed thematically for dominant themes using the constant comparative method.

## 3. Results

A total of 63 participants were enrolled in the study, of which 36 (57%) completed the entire eight weeks of the Heartfulness meditation program. One participant dropped out due to a COVID-19 infection in the family and another due to personal non-COVID-19-related health issues. The remaining dropped out participants reported a lack of time commitment. Among 36 participants who completed 8 weeks of the program, 31 (86%) were female. While one participant did not complete the demographic information sheet, 11/35 (31%) were healthcare professionals, and 24/35 (69%) were non-healthcare professionals. 

### 3.1. Adherence to Practice 

A total of 28 out of 36 participants (77.7%) attended at least two group trainer-guided meditation sessions per week. There is no acceptable minimum adherence level in clinical trials regarding Heartfulness meditation self-practice. If participants practiced any of the suggested self-practices (Meditation, Rejuvenation, and bedtime prayer meditation) four or more times a week, it was considered adherence to suggested self-practice for our study. A total of 18 out of 31 participants (58.1%) adhered to the suggested self-practice regimen. Five participants did not report adherence data regarding self-practice. In addition, on average the participants completed approximately eight HeartsApp meditation sessions across the duration of the program. 

Weekly results in the text below and throughout are expressed as (mean ± standard error).

### 3.2. Survey on COVID-19-Related Stress 

There was a significant interaction between Time and Health Care Professional status, so all pairwise comparisons between Time and Health Care Professional status were made. There was a significant decrease in worry about a family member experiencing a COVID-19 infection for non-healthcare professionals between week 0 (6.40 ± 0.45) and week 4 (5.12 ± 0.49) (*p* = 0.0014), as well as between week 0 (6.40 ± 0.45) and week 8 (5.06 ± 0.50) (*p* = 0.0009). There were no significant differences in worry about a family member experiencing a COVID-19 infection for healthcare professionals. There were no significant differences in worry about a family member experiencing a COVID-19 infection between healthcare professionals and non-healthcare professionals at any of the time points. There were no significant differences in worry about participants themselves experiencing a COVID-19 infection for healthcare professionals or non-healthcare professionals.

### 3.3. Effect on Stress (PSS)

The results of Tukey’s multiple comparison procedure for PSS are given below in Table 1. There was a significant decrease in perceived stress between week 0 (20.76 ± 0.70) and week 4 (14.97 ± 0.87) for all subjects regardless of Health Care Professional status (*p* < 0.0001). As shown in Table 1, the estimated mean difference was 5.79 points lower at week 4. There was also a significant decrease in perceived stress between week 0 (20.76 ± 0.70) and week 8 (14.09 ± 0.84) for all subjects regardless of Health Care Professional status (*p* < 0.0001). The estimated mean difference was 6.68 points lower at week 8. There was insufficient evidence to suggest any difference in perceived stress between week 4 and week 8 (*p*-value = 0.53).

### 3.4. Effect on Sleep Quality (PSQI)

The results of Tukey’s multiple comparison procedure for PSQI are given below in Table 2. There was a significant decrease in PSQI between week 0 (8.66 ± 0.42) and week 4 (7.11 ± 0.51) for all subjects, regardless of Health Care Professional status (*p* = 0.0022). The estimated mean difference was 1.55 points lower at week 4. There was also a significant decrease in PSQI between week 0 (8.66 ± 0.42) and week 8 (6.61 ± 0.50) for all subjects, regardless of Health Care Professional status (*p* < 0.0001). The estimated mean difference was 2.05 points lower at week 8. There was insufficient evidence to suggest any difference in PSQI between week 4 and week 8 (*p*-value = 0.54).

### 3.5. Qualitative Thematic Analysis 

A total of 21 participants volunteered to participate in an optional interview, of which 17 (80.95%) participants were planning to continue Heartfulness meditation, and 15 (71.43%) participants were either planning to or already recommended these meditation practices to others. The qualitative analysis of narratives describing the impact of Heartfulness meditation on overall wellbeing, interactions with others, and outlook of life identified several themes. Table 3 describes the major themes along with their description and examples of comments from the participants. Primarily, participants suggested that their attitude changed to be more positive, empathetic, and disciplined. Specifically, in coping with the COVID-19 pandemic, one participant said, “It [Heartfulness meditation practices] made me feel safer, less helpless and more hopeful”. Other comments from participants are shown in Table 3. 

While specific comments are included in Table 3, one participant, who took anxiety medication in the past (stopped two years before participating in the study), suggested that Heartfulness meditation practices helped them feel less anxious and less stressed more naturally. Some participants also suggested that these meditation practices alleviated stress and made them calmer and more focused. Moreover, some participants talked about how these meditation practices helped alleviate stress and improve sleep. Below are the specific comments from the participants:

“I have less fatigue because I feel my sleep has improved due to meditation”.

“I think Heartfulness has had an impact on my long intermittent insomnia. When I wake up anywhere between 2:30–5:30 a.m. [now], I feel calm and confident that I will fall back asleep whereas pre-Heartfulness I felt anxious and most often did not fall back asleep”.

Participants also talked about how these practices improved their ability to control emotional reactions and accept their circumstances. Specific comments are included in Table 3. One participant summed up all the effects and themes and said, “One impact is that when I have been doing the [Heartfulness meditation] sessions, it brings a calming effect to me. Additionally, I think that trickles over into the rest of your life, your interactions with people, and just to being thoughtful and mindful in how I am interacting with people, and I think it’s somewhat generated from the peace within”.

## 4. Discussion

Stress and lack of quality sleep are the two most essential precursors leading to chronic medical and mental health problems influencing the health of our community. Several studies depict many stress-related health problems related to disasters or public health crises, including mental health problems, substance abuse, suicide, sleep disturbances, and cardiovascular disease [15,16]. In current times, COVID-19 pandemic has disrupted virtually every aspect of daily living and created an unprecedented stressful situation for all of humanity around the globe. Several studies have reported higher levels of stress, anxiety, and depression, among the general population and healthcare workers because of the COVID-19 pandemic since December 2019 [17,18,19,20,21]. This study also highlights the burden of stress and poor sleep quality, affecting healthcare and non-healthcare workers equally during the COVID-19 pandemic. A public health crisis can have both immediate and long-lasting adverse effects on the health and wellbeing of people. Our study using the mixed method, i.e., quantitative and qualitative measures, reports the impact of Heartfulness meditation practice on psychological wellbeing during the COVID-19 pandemic. 

Meditation programs are conventionally conducted in in-person settings, and it is conceivable that it may be harder to engage in a virtual environment than in-person sessions. A recent study evaluated the effects of mindfulness training using similar PSS and PSQI measures as our study. They found that virtual mindfulness training was equivalent to in-person mindfulness training to reduce stress during and before the pandemic. However, they did not find evidence that this training improved sleep quality [13]. In another study, an 8-week mindfulness-oriented meditation (MOM) course where 75% of the course was conducted virtually showed an improvement in the psychological wellbeing of female teachers during the COVID-19 pandemic [14]. A recent study showed that a brief, virtual, Heartfulness meditation program via audio relaxation techniques through a Heartfulness trainer improved sleep quality and loneliness among physicians and advance practice providers [7]. Our study showed that following Heartfulness meditation practice, PSS and PSQI improved significantly in the participants from different parts of the United States. About 31% were healthcare professionals, and the entire program was conducted virtually. Based on these observations, we propose that meditation programs offered via virtual platforms can offer a convenient, helpful, and easily accessible tool to a large community at once to help improve the psychological wellbeing of individuals. 

This study adds to the existing literature supporting the benefits of Heartfulness practice, as reported by some previous studies showing the benefit of Heartfulness relaxation and meditation to reduce stress, burnout, loneliness, and improves the quality of sleep [7,8,9,22]. The exact mechanism of Heartfulness meditation is not well understood. The results of qualitative analysis in our study bring a unique perspective to this aspect as we were able to show that the participant’s subjective experiences strongly supported the results of the survey findings. Thus, these results enhance our understanding of how Heartfulness meditation practice helps reduce stress and improve the quality of sleep. Our qualitative analysis suggests these effects could be because a simple heart-based meditation brought a ‘calming effect’ in our participants, resulting in ‘inner peace’. Such an effect also resulted in inner changes in our participants, including positive thinking, accepting and empathic attitude, and an increase in awareness of one’s own emotions and the needs of others. 

We noted a significant decrease in worry about a family member experiencing a COVID infection for non-healthcare professionals but not among healthcare professionals. This difference could have been due to healthcare professionals having a more apprehensive perception of COVID-19 infection and its severity while serving the affected patients. Interestingly, we found no significant change in worry of participants experiencing a COVID-19 infection themselves during the study period even though there was a significant improvement in quality of sleep and level of stress. Regarding this observation, we speculate based on qualitative analysis that Heartfulness meditation practice was likely helpful in cultivating resilience and coping with difficult circumstances in a calmer state of mind, resulting in the reduction in stress.

The guided relaxation that preceded every group meditation session was also very beneficial in reducing stress and anxiety and helped participants practice meditation more effectively. The guided relaxation likely stimulates the parasympathetic pathway in the brain, which helps establish a relaxed state of mind before meditation.

This study has several limitations. The sample size was relatively small. As this was a pretest–posttest design, there was a lack of a control group, and participants in the study self-selected meditation, suggesting the possibility of selection bias. We also cannot exclude the possibility of unknown sources of bias and the personal life circumstances of participants that could have played a role in the changes noticed. Of the 63 enrolled participants, 36 completed the study, indicating a high attrition rate of 43%. Most of the participants dropped out within the first three weeks and reported an inability of time commitment. The dropouts could have served as a control group if we had administered the post-intervention surveys. Similar high attrition rates were observed in previous studies among the intervention group [7,23,24]. An additional perceived limitation could be the use of personal reporting of self-practice of the different meditation techniques and variability in individual adherence. However, we recorded attendance in virtual group meditation sessions conducted by the Heartfulness trainer to accurately analyze adherence with the primary requirement of the program. Another limitation of the study is that our data may not include the effect of the passage of time on perceived stress and sleep quality. However, the surveys and subsequent interviews specified that the focus of interest was their experience with the meditation sessions and practices throughout the study period. Additionally, in survey studies relying on human recollection, the responses may be imprecise. Long-term data about the sustained improvement in sleep quality and reduction in stress need to be explored in future studies.

## 5. Conclusions

Virtually accessible meditation programs such as Heartfulness practice could be an effective tool to offer to a large community at once. Such a practice could be feasible to incorporate into the current lifestyle of many individuals, regardless of one’s vocation. Our study demonstrated statistical improvements in perceived stress score and sleep quality index in participants undergoing a virtual Heartfulness meditation program. Heartfulness practice may also help enable individuals to cultivate a quality of empathy, acceptance, and individual peace. Promoting healthier lifestyles, including meditation practice, can be an essential preventive measure to combat possible future health problems related to psychological stress. The effects of virtually accessible Heartfulness meditation practice need to be explored further in larger studies.

## Figures and Tables

**Table 1:** Results of Tukey’s multiple comparison procedure for PSS.

Comparison	Mean Difference	*p*-Value	95% C.I.
Week 0–Week 4	5.79	<0.0001	(3.94, 7.65)
Week 0–Week 8	6.68	<0.0001	(4.89, 8.47)
Week 4–Week 8	0.89	0.53	(−1.09, 2.86)

**Table 2:** Results of Tukey’s multiple comparison procedure for PSQI.

Comparison	Mean Difference	*p*-Value	95% C.I.
Week 0–Week 4	1.55	0.0022	(0.49, 2.61)
Week 0–Week 8	2.05	<0.0001	(1.03, 3.07)
Week 4–Week 8	0.50	0.54	(−0.62, 1.62)

**Table 3:** Emerging themes on the impact of the Heartfulness meditation program.

Theme	Description	Example Comment from Respondents
The attitude changed to be more Positive thinking, Empathetic, and Disciplined.	The attitude towards others and life changed positively after participating in the study. Participants suggested they became more compassionate, understanding and started thinking positively. They became more disciplined as well.	“I feel a bit more patient; I feel a little bit more compassionate, a little bit more open to other people, and less judgmental.” “I find that I am just in general a lot more thankful about very simple things.” “I noticed that I reconnected to some of the human side of things. Because sometimes we can get lost in the process and the thinking about the whole thing, you sort of forget that is actually a person.”
Alleviated stress and made calmer and more focused	The practice helped alleviate stress, and participants felt more focused and calmer	“I found the meditation to be extremely helpful for just staying calm in the storm, but I did not grow up with that sort of understanding.” “I was having a harder time concentrating, especially on projects that required a lot of thought. I really dreaded them because I just had a hard time staying focused. Additionally, that has been a big improvement for me because of the meditation I have been doing. It has helped me a lot and I do not dread those projects.”
Ability to control emotional reactions and became more mindful and accepting	The practice helped develop the ability to control emotional reactions. Some participants suggested they became more mindful of the situations and accepting	“I have been able to listen better. Even if what I am listening to contradicts my own personal views. I listen, I do not react, and I do not respond with the intensity that I want to—instead I accept them and move on.” “I am now mindful of the fact that I am always in a hurry and avoid unplanned interactions with neighbors and friends. So, I am trying to work on slowing down and allowing time for spontaneous phone conversations or physical interactions.” “It was like, if bad things are happening, of course, you are going to feel bad and react badly. Now [after meditation practices] it’s like, I actually have the power in this to stay calm and observe both emotions in my own self, and then things around me and that was just very very helpful.”

## Data Availability

Data Availability Statement: Data available upon request from the corresponding author.
